# Reproducible EEG-BCI Experiments: Frame-Confirmed Timing and Physical SSVEP Validation

**DOI:** 10.3390/s26217065

**Published:** 2026-11-08

**Authors:** Janis Peksa, Luigi Bianchi

**Affiliations:** 1 Institute of Information Technology, Riga Technical University, LV-1658 Riga, Latvia; 2 Department of Civil Engineering and Computer Science Engineering, University of Rome Tor Vergata, 00133 Rome, Italy; luigi.bianchi@uniroma2.it

**Keywords:** brain–computer interface, EEG, SSVEP, reproducibility, stimulus timing, experimental provenance

## Abstract

Reproducible EEG-based brain–computer interface (BCI) experiments require traceability from the configured protocol through runtime execution to acquisition context and, where relevant, the physical stimulus delivered to the participant. This study presents and evaluates a workflow that links machine-readable SSVEP protocols, deterministic session planning, frame-confirmed events, raw acquisition provenance, post-session integrity checks, and independent photoresistor-based display validation. The contribution is not a new acquisition platform or timing sensor, but the integration of these evidence layers into a single auditable experimental chain and the empirical assessment of their consistency. All 61 automated tests passed for the frozen software release. Three optical sweeps covering 8–15 Hz yielded 48 valid measurements, all within the predefined ±0.25 Hz criterion. The mean absolute error was 0.0345 Hz, and the maximum was 0.0668 Hz. In a separate 12.75 Hz rendering-load experiment, all 12 measurements also passed, with mean absolute errors of 0.0170 Hz for a single stimulus and 0.0154 Hz with three simultaneous distractors. Local optical pulse-width anomalies were, nevertheless, observed, particularly at 13–14 Hz and in two multi-stimulus repetitions, showing that dominant-frequency agreement and local timing regularity are distinct validation properties. Formal empirical validation targeted software and session integrity and physical display output; it did not quantify end-to-end event-to-EEG latency through the OpenBCI transport path. The results support a more explicit separation of configured intent, runtime timing evidence, acquisition provenance, and physical stimulus verification in reproducible EEG-BCI experimentation.

## 1. Introduction

Brain–computer interfaces (BCIs) translate measured brain activity into information that can be used by an external computational system. In electroencephalography (EEG)-based BCIs, however, the recorded signal and its eventual interpretation are shaped by more than the decoding algorithm. Acquisition hardware, electrode placement, experimental paradigm, stimulus generation, event timing, signal processing, and software architecture all contribute to the conditions under which data are produced. This diversity has enabled motor imagery, evoked potential, steady-state visual evoked potential (SSVEP), cognitive state, and other paradigms, but it has also produced substantial methodological heterogeneity across implementations [1]. An EEG-BCI experiment is, therefore, better understood as a chain of interdependent experimental and computational steps than as a recording followed by classification.

Reproducing such an experiment requires enough information to reconstruct both the analysis and the conditions under which the data were acquired. This means comprehensively documenting the hardware and software environment, including the specific EEG device, channel montage, reference arrangement, and sampling characteristics, alongside experimental parameters like stimulus definitions, trial structures, event timing, and preprocessing settings. A recent systematic review identified heterogeneous acquisition settings, incomplete metadata, inconsistent preprocessing descriptions, dataset fragmentation, and subject- and session-related variability as connected reproducibility problems in EEG-BCI research [2]. EEG-BIDS addresses an important downstream part of this problem by standardizing EEG dataset organization and metadata [3], while the Mother of all BCI Benchmarks (MOABB) provides standardized interfaces and pipelines for reproducible evaluation across public BCI datasets [4]. These initiatives demonstrate the value of explicit structure and metadata, but they do not by themselves establish what occurred at stimulus delivery and acquisition time in an individual experiment.

Existing BCI software ecosystems address complementary parts of the experimental workflow. BCI2000 integrates signal acquisition, processing, experimental control, and BCI applications within a general-purpose architecture [5]. OpenViBE provides an open environment for designing and operating BCI experiments and real-time processing scenarios [6], while Lab Streaming Layer (LSL) supports timestamped and synchronized streaming across heterogeneous neural, physiological, behavioral, and environmental sources [7]. These systems are important foundations for reproducible experimentation, yet reproducibility still depends on preserving the correspondence between the configured experiment, the sequence that actually ran, the acquisition context that was recorded, and physically meaningful conditions experienced by the participant.

Computer-generated visual stimulation makes this correspondence particularly important. The instant at which software requests a visual event does not necessarily coincide with the event emitted by the monitor. Operating-system scheduling, graphics drivers, rendering load, synchronization with the refresh cycle, and display electronics can affect presentation timing. Studies of PsychoPy and other visual experiment generators show that high timing accuracy is achievable but remains dependent on the specific hardware and software configuration [8,9]. Large comparative timing studies, therefore, recommend validating the actual stimulus and computer configuration rather than relying on nominal software timing alone [9]. External optical measurement provides a direct route for testing whether a configured timing or frequency parameter was physically realized by the display.

SSVEP-based BCIs are well suited to this type of validation because the experimental label has a direct physical interpretation. Repetitive visual stimulation elicits EEG components at the stimulation frequency and its harmonics, and frequency recognition methods explicitly use that relationship. Canonical correlation analysis, for example, identifies SSVEP responses by correlating EEG with reference signals constructed from candidate stimulus frequencies [10]. A label such as 13 Hz, therefore, denotes an intended physical condition rather than an arbitrary class name. If the display produces a different dominant frequency or irregular local timing, that discrepancy becomes part of the experimental provenance.

Open and relatively inexpensive EEG hardware has made experimental BCI systems easier to construct. OpenBCI Cyton provides eight acquisition channels at 250 Hz and has been used in recent SSVEP studies with occipital and parietal montages [11]. BrainFlow provides a hardware-independent biosensor acquisition interface that can support OpenBCI and other devices [12]. At the processing level, contemporary SSVEP workflows include task-discriminant component analysis [13], filter-bank canonical correlation analysis [14], and related spatial-filtering approaches [15], while the BRAInDA project provides reusable BCI datasets and algorithm implementations [16]. These resources improve acquisition and analysis reproducibility, but they do not remove the need to retain acquisition-time evidence about how a configured stimulus was executed and what physical output was actually produced.

Recent BCI frameworks reinforce this distinction. Cardona-Álvarez et al. developed an OpenBCI framework supporting distributed acquisition, stimulus delivery, flexible electrode configuration, and marker synchronization [17]. Gemborn Nilsson et al. proposed a hardware-independent human-in-the-loop framework combining real-time stimulus control, LSL communication, model training, and online classification [18]. These systems demonstrate that integrated acquisition and experimental-control environments are established practice. The present problem is, therefore, not the absence of BCI software frameworks, but the narrower question of how experimental intent, runtime confirmation, acquisition provenance, and independently measured physical stimulus output can be retained as distinct but linked evidence.

Reproducibility work at the analysis and benchmarking stages has also advanced considerably. A recent large-scale MOABB study reimplemented 30 machine learning pipelines across 36 public BCI datasets, including SSVEP datasets, to establish open and comparable benchmarks [19]. SpeechBrain-MOABB extends this direction toward reproducible deep learning pipelines [20], while cross-software reproduction studies show that nominally equivalent EEG preprocessing workflows can still produce measurable differences across software environments [21]. These studies address reproducibility after data have been collected. They do not directly test whether the experimental condition encoded in the dataset corresponds to the runtime and physical condition delivered during acquisition.

Physical stimulus verification is likewise established rather than novel in itself. Photodiode measurements have been used in SSVEP research to verify flicker timing and frequency and to provide positive controls for spectral analyses [22]. The FreqTag toolbox emphasizes accurate stimulus timing and the dependence of frequency-tagging paradigms on display characteristics [23], while experimental work has shown that the monitor refresh rate can materially influence steady-state visual stimulation [24]. More recent lifecycle-oriented environments such as NeuronLab also integrate multiple phases of BCI experimentation and analysis [25]. Taken together, the literature shows that acquisition frameworks, reproducible analysis, metadata standards, and optical validation each address important parts of the problem; however, these practices are usually treated as separate layers.

The research gap addressed here is, therefore, not the absence of any one of these established techniques. It is the lack, within the tested low-cost EEG-BCI workflow, of an explicitly auditable chain that preserves the correspondence among four evidence layers: configured experimental intent, runtime-confirmed stimulus execution, acquisition and session provenance, and independently measured physical stimulus output. This distinction is scientifically relevant because agreement at one layer does not guarantee agreement at another. In particular, a display can preserve the expected long-window dominant SSVEP frequency while still exhibiting local pulse-width irregularities that a frequency-only check would not reveal.

To examine this gap, the present study evaluates a reproducibility-first workflow in which machine-readable configuration is carried into deterministic session planning, frame-confirmed runtime events, raw acquisition records, provenance artifacts, automated integrity checks, and independent optical SSVEP validation. SSVEP stimulation was selected because stimulus frequency can be specified in software and recovered independently from display luminance. Configured frequencies from 8 to 15 Hz were evaluated, followed by a rendering-load test of a fixed 12.75 Hz target. The formal empirical evidence concerns software and session integrity and physical stimulus delivery; participant-level classification accuracy and end-to-end event-to-EEG hardware latency are outside the validated scope of this study.

Four research questions guided this study and operationalized this scope:

• RQ1 asks whether a configuration-driven EEG-BCI workflow can maintain an auditable link between a predefined protocol and the artifacts produced during execution;
• RQ2 concerns whether frame-confirmed events and acquisition provenance are sufficient for automated post-session integrity checks without reconstructing experimental meaning from EEG sample indices alone;
• RQ3 examines the agreement between configured and physically measured SSVEP frequencies across 8–15 Hz and the stability of the measured signal during concurrent rendering;
• RQ4 asks whether the acquisition, recording, processing support, and physical validation components can be verified reproducibly from a frozen software release and its automated test suite.

The contribution is methodological rather than algorithmic. The individual components used here, which include machine-readable configuration, BCI acquisition frameworks, frame-aware visual presentation, provenance records, integrity checks, and optical stimulus measurement, are established practices and are not claimed as individually novel. The contribution lies in integrating them into a single auditable experimental chain and empirically testing consistency across distinct evidence layers. The principal empirical observation is that accurate long-window dominant SSVEP frequency can coexist with local optical timing irregularities, supporting the need to retain frequency accuracy and local timing diagnostics as separate validation evidence. This work does not introduce a new SSVEP decoder, EEG headset, data standard, or general-purpose BCI framework.

Importantly, the contribution should not be interpreted as the mere aggregation of established technical practices. The scientific question examined here is whether independently recorded evidence layers describing the same experimental condition remain mutually consistent under a frozen implementation, and whether disagreement between those layers can expose properties that would remain hidden when only one form of validation is considered. The experiments provide a concrete example: dominant-frequency agreement can coexist with local physical timing irregularity. The methodological contribution is, therefore, the explicit formulation and empirical evaluation of cross-layer consistency within the experimental provenance chain.

The remainder of this paper is organized as follows. Section 2 describes the configuration, acquisition, provenance, processing support, and independent optical validation procedures. Section 3 reports the software verification, session integrity, frequency accuracy, and rendering load results. Section 4 discusses the findings relative to existing BCI frameworks and reproducibility practices, together with the study limitations, and Section 5 summarizes the conclusions.

## 2. Materials and Methods

### 2.1. Study Design and Reproducibility Requirements

This study was designed as a technical and methodological validation of a reproducibility-oriented EEG-BCI workflow. Rather than comparing classifiers or estimating population-level BCI performance, this study examined whether the experimental chain preserved an auditable link between predefined conditions, runtime stimulus execution, EEG acquisition context, session provenance, and independently measured stimulus properties.

Six requirements guided the design. The experimental protocol and hardware configuration had to be represented explicitly in machine-readable form rather than only in procedural code. Each validated configuration had to generate a deterministic session plan with explicit trial and presentation identities. Visual stimulus onset and offset had to be tied to runtime events confirmed at the display frame-swap boundary. Raw acquisition had to remain separate from optional online processing, and each session had to retain enough provenance for automated consistency and integrity checks. Finally, when an experimental parameter had direct physical meaning, as SSVEP frequency does, the delivered condition had to be measurable independently of its configured software value.

The empirical validation focused on software integrity, session integrity, and physical stimulus delivery. Previously recorded development or pilot EEG sessions were not included in the analyses reported in this study, and no population-level claims were made about SSVEP classification accuracy. The optical validation did not involve human participants. Table 1 summarizes the six requirements and the corresponding implementation mechanisms.

### 2.2. Software Architecture

The repository contains separate components for SSVEP experimentation, physical stimulus validation, and an additional imagined-speech acquisition workflow. Two components are evaluated here. The ‘ssvep-bci’ package implements stimulus presentation together with acquisition, recording, processing support, and verification. The ‘ssvep-frequency-test’ package provides an independent Arduino and photoresistor workflow for measuring the visual stimulation produced by the display.

The ‘ssvep-bci’ package requires Python 3.11 or later and is organized into modules for configuration loading, experiment planning, runtime control, visual rendering, EEG acquisition, event handling, session recording, digital signal processing, model training, verification, and the user interface. Visual stimuli are rendered with PySide6 through a ‘QOpenGLWidget’. The OpenGL surface requests a swap interval of one to synchronize buffer swaps with the display refresh cycle. At startup, the application queries the selected display refresh rate and rejects configured stimulus frequencies at or above one-half of that rate.

EEG acquisition runs separately from visual presentation and can use interchangeable backends. The repository includes BrainFlow [12], Lab Streaming Layer (LSL) [7], and synthetic acquisition. BrainFlow supports the OpenBCI hardware configuration, while the synthetic backend allows deterministic software and display validation runs without a participant or physical EEG device.

The ‘ssvep-frequency-test’ component is independent of the SSVEP presentation process. It records the photoresistor output through an Arduino and later aligns the optical trace with structured stimulus events written by the SSVEP application. The presentation program neither starts nor controls the photosensor recorder, so the system that generates the visual stimulus remains separate from the system that measures it.

**Figure 1:**
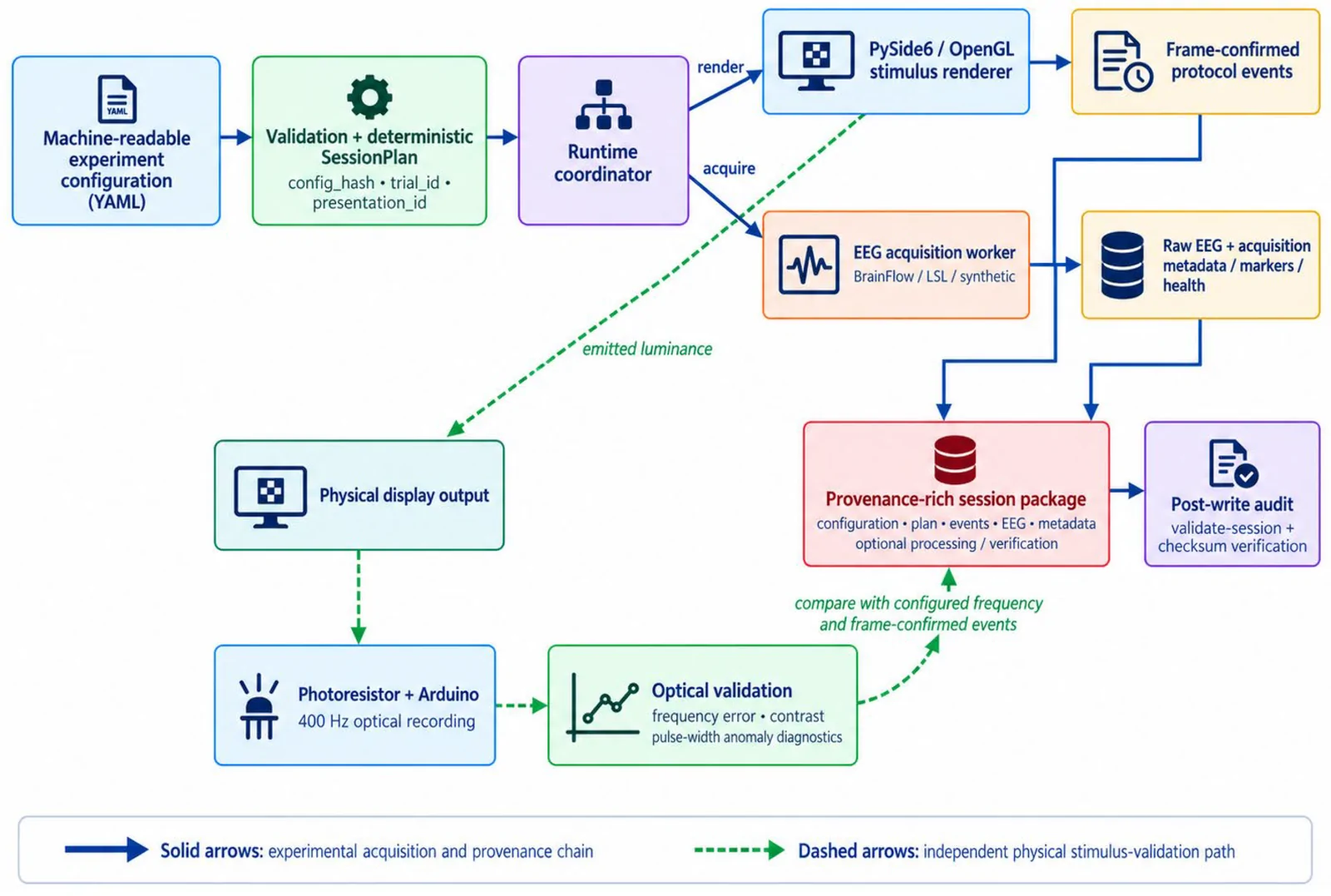
Reproducibility-first architecture of the EEG-BCI experimental workflow. Machine-readable experimental configuration is validated and compiled into a deterministic session plan containing configuration, trial, and presentation identities. During execution, visual stimulation and EEG acquisition produce independently recorded runtime evidence. Visual events are acknowledged at the frame-swap boundary, whereas raw EEG and acquisition metadata are preserved independently of optional processing. These records are combined into a provenance-rich session package subject to post-write integrity validation. The dashed branch represents an independent physical validation path in which emitted monitor luminance is recorded with a photoresistor–Arduino system and analyzed against configured stimulus parameters and frame-confirmed events.

A separate imagined-speech acquisition application is also present in the repository, but it was not evaluated in this study, and no imagined-speech decoding results are reported. It is mentioned only to show that the broader acquisition architecture is not restricted to the SSVEP paradigm. Figure 1 summarizes the architecture considered in this paper.

### 2.3. EEG Hardware and Acquisition Configuration

The supported SSVEP hardware configuration used an OpenBCI Cyton board with an eight-channel occipital and parietal montage sampled at 250 Hz. The configured EEG positions were O1, O2, Oz, PO3, PO4, P3, P4, and Pz. The device profile specified linked mastoids as the reference and the Cyton BIAS connection as ground. Channel values were recorded in microvolts.

For the BrainFlow backend, the software verified at connection time that the sampling rate reported by the board matched the 250 Hz rate specified in the device profile. The configured channel source indexes were additionally checked against the EEG rows reported by BrainFlow. When automatic serial-port selection was used, exactly one FTDI- and OpenBCI-compatible serial device was required to be present.

BrainFlow timestamps were treated as source timestamps in Unix epoch seconds. At acquisition initialization, an offset between wall-clock and host monotonic time was estimated and used to obtain aligned monotonic timestamps for incoming EEG samples. Each recorded sample retained the source timestamp, corrected source timestamp, aligned monotonic timestamp, host receipt time, EEG channel values, and, where available, an embedded acquisition marker. This alignment places software-side acquisition timestamps and frame-confirmed protocol events in a common host monotonic clock domain for post-session comparison. It does not, however, constitute a measurement of physical display-to-EEG latency, OpenBCI transport latency, or marker insertion latency.

The frozen validation series was executed on Microsoft Windows 10 Pro (build 19044) using a host computer with an Intel Core i7-11700F processor, 31.8 GB of RAM, and an NVIDIA GeForce RTX 3050 GPU (driver 32.0.15.6094). Visual stimulation was presented full-screen on an HP P27q G4 monitor at 2560 × 1440 pixels, 100% Windows scaling, and a Windows-reported refresh rate of 59 Hz (nominal panel range: 50–60 Hz). These display settings were held constant throughout the formal optical validation series.

### 2.4. Machine-Readable Experiment Configuration

Experiments were defined using YAML configuration files validated against strict data models at application startup. The configuration represented the experiment identifier, random seed, device profile, protocol timing, visual stimuli, presentation parameters, output settings, processing configuration, marker codes, and external resources.

Stimulus definitions included a stable stimulus identifier, target frequency, phase offset, duty cycle, and visual geometry. Visual geometry specified shape, dimensions, normalized screen location, and on/off colors. Protocol configuration specified the sequence of stimuli, whether multiple stimuli were presented simultaneously, the number of repetitions, initial and final rest periods, prestimulus duration, stimulation duration, intertrial interval, and optional sequence breaks.

Paths to device profiles, external resources, and classifier artifacts were resolved relative to the configuration file and validated before experiment execution. The loader also checked consistency between processing channel definitions and the selected acquisition device and rejected processing configurations whose frequency or harmonic settings were incompatible with the acquisition Nyquist frequency.

For every session, the effective experiment configuration and device profile were copied into the session directory. They were retained as part of the session record rather than used only as transient launch settings.

### 2.5. Deterministic Session Planning and Experimental Identity

Before execution, each validated experiment configuration was compiled into a SessionPlan. Trial randomization, where enabled, used the explicit random seed contained in the configuration. A SHA-256 fingerprint was calculated from the normalized experiment configuration, and this configuration hash was used to derive the session plan identifier.

The compiled plan contained an ordered sequence of steps representing initial rest, prestimulus periods, stimulus presentations, intertrial periods, and final rest. Each trial received a stable trial_id, while each actual visual presentation received an independent presentation_id. These identifiers were subsequently propagated into runtime event records and processing windows.

Keeping the configuration, compiled plan, trial identity, and presentation identity separate preserves the link between experimental intent and the sequence that actually ran. It also avoids downstream dependence on implicit assumptions about trial order or the numerical location of samples within a single EEG file.

### 2.6. Visual Stimulation and Frame-Confirmed Events

Visual SSVEP stimuli were rendered by a PySide6 QOpenGLWidget. During flashing, the renderer calculated stimulus phase from elapsed monotonic time according to the configured frequency and phase offset. For a square-wave stimulus with duty cycle d, the displayed state was ON when the fractional cycle phase was smaller than d and OFF otherwise. The configurations used in the present study specified a duty cycle of 0.5.

The application distinguished between a request to begin or end a presentation and confirmation that the corresponding visual state had reached the OpenGL frame-swap boundary. When flashing was requested, the renderer marked an onset acknowledgement as pending. The first subsequently swapped frame on which the target stimulus had been painted in the ON state generated a PresentationFrameAcknowledged command. The monotonic time of this acknowledgement became the recorded stimulus-onset timestamp and reset the phase anchor used for subsequent flickering.

At stimulus termination, an offset acknowledgement was generated after a swapped frame on which the flashing stimulus was no longer painted in the ON state. The runtime then emitted structured stimulus_onset and stimulus_offset protocol events with the payload field confirmed_by_frame_swap = true. If a session was aborted before a confirmed offset, the generated offset record was explicitly marked as not frame-confirmed.

Frame-swap confirmation was used only as evidence that the intended display state had reached the software or display-swap boundary. It was not assumed to represent the exact luminance waveform emitted by the monitor. Physical output was measured separately with the photoresistor procedure described in Section 2.10, Section 2.11 and Section 2.12.

Stimulus phase was computed from elapsed host monotonic time rather than advanced by counting rendered frames. A delayed, repeated, or missed display update, therefore, does not permanently accumulate phase error in subsequent software states. Consequently, a long-window Fourier estimate can remain close to the configured target frequency even when local optical pulse widths are irregular. Frame confirmation reduces ambiguity between a presentation request and the first or last acknowledged buffer swap, but it does not by itself establish the exact time of emitted luminance or synchronization to a physical EEG sample.

### 2.7. EEG Acquisition, Markers, and Raw Data Preservation

The acquisition subsystem owned the selected hardware backend on an independent worker thread. Backend operations were serialized on this thread, and incoming sample batches were passed to a separate raw-data writer. Experimental markers were likewise queued and inserted through the acquisition backend where supported. Marker insertion attempts were recorded independently, including the associated event identifier, event sequence number, marker code, insertion attempt timestamp, backend support status, success status, and error information where applicable.

Raw EEG was written asynchronously to eeg_raw.csv. The file contained, for every sample, a sequential sample index, host receipt monotonic timestamp, host receipt wall-clock timestamp, original source timestamp, corrected source timestamp, aligned monotonic timestamp, the configured EEG channels, and any embedded hardware marker. The writer used a bounded queue; queue overrun or writing failure was treated as an acquisition or session error rather than silently discarding samples.

The acquisition architecture records marker insertion attempts and sample index continuity so that transport and recording problems can be detected when they occur. However, the formal optical validation experiments reported in this study used the synthetic acquisition backend. They, therefore, did not provide empirical estimates of end-to-end event-to-EEG alignment, OpenBCI marker latency, or packet-loss characteristics on a physical OpenBCI link. These quantities remain implementation- and hardware-specific validation targets outside the present formal series.

Online processing, when enabled, was kept separate from the raw-data writer and saved to ‘processing-results.jsonl’. The raw EEG record remained unchanged by filtering, epoch extraction, or classification.

### 2.8. Session Provenance and Automated Integrity Validation

Each run produced a dedicated session directory containing the experiment and device configurations, compiled plan, structured runtime events, raw EEG, acquisition metadata, marker outcomes, health records, optional processing or verification outputs, and optional SHA-256 digests. Table 2 lists these artifacts and their role in the reproducibility record.

**Figure 2:**
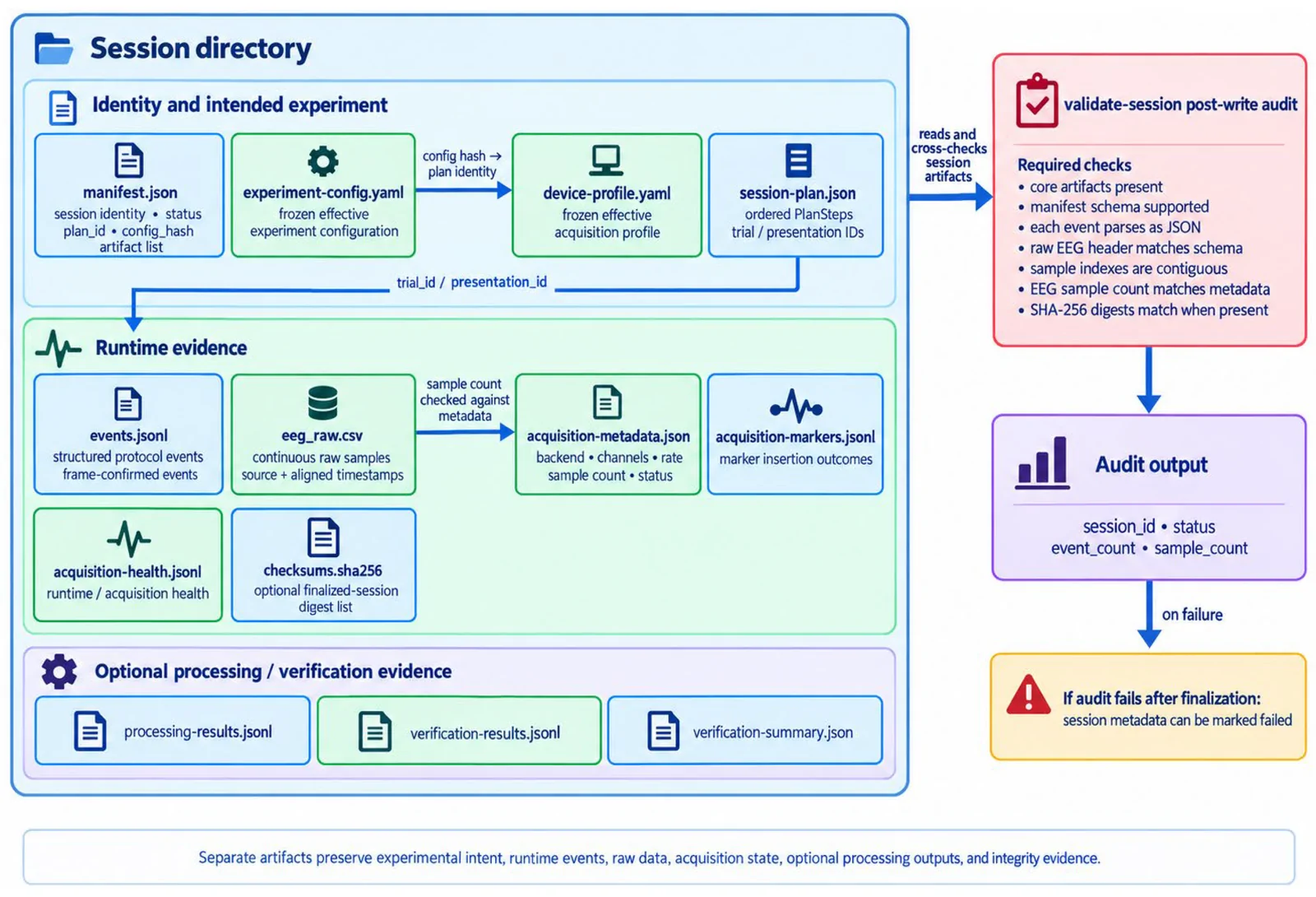
Provenance structure of an SSVEP session and post-write integrity validation procedure. The session directory separately preserves experiment and device configurations, the compiled execution plan, runtime events, raw EEG, acquisition metadata, marker and health information, optional processing and verification outputs, and optional SHA-256 digests. The validate-session procedure verifies the presence of required core artifacts, manifest compatibility, event-record syntax, raw EEG structure and sequential sample indexes, agreement between the recorded and metadata sample counts, and artifact digests when checksum information is available. A failed post-write audit can cause finalized session metadata to be marked as failed rather than leaving an apparently valid but internally inconsistent recording.

The ‘validate-session’ procedure performs a post-write integrity audit. It requires all core artifacts to be present, verifies the manifest schema, parses each non-empty protocol-event record as JSON, checks the raw EEG header, requires sample indices to remain contiguous from zero, and compares the number of EEG samples with the count stored in acquisition metadata. For finalized sessions with checksums, each SHA-256 digest is recalculated and compared with the recorded value. A session that fails this audit can be marked as failed rather than retained as apparently valid. Figure 2 shows the provenance structure and the audit relationship.

For this study, session integrity behavior was established through the frozen automated test suite rather than through a separate archived reference-session audit. The tests covered complete synthetic-session recording, required raw-session structure, frame-acknowledged stimulus intervals, and manifested and checksummed verification artifacts. For the formal optical series, the archive also retained the source session identifier, profile, UTC creation time, frozen software revision, and terminal status. The complete session directories themselves were not included in the archived validation package analyzed here.

### 2.9. Reference SSVEP Processing and Verification Workflow

No external or public EEG dataset was used as empirical evidence in this study. The formal quantitative dataset consisted of the optical validation recordings and the associated software test and provenance artifacts described in Section 2.10, Section 2.11, Section 2.12, Section 2.13, Section 2.14 and Section 2.15. An optional SSVEP processing route is documented here only to show how the same acquisition and provenance records can be carried into participant-specific EEG analysis without redefining trial semantics. Classification performance itself was not a primary outcome.

The reference collection configuration used four simultaneously visible 200 × 200-pixel square stimuli at 8, 9, 13, and 14 Hz. The target stimulus was indicated by a visual cue, while all four stimuli remained available during the stimulation interval. The acquisition configuration used the eight occipital and parietal channels described in Section 2.3. A standard collection session contained 12 repetitions of each class, with 4 s stimulation periods, 1 s prestimulus periods, and 3 s intertrial intervals.

Participant-specific FBTDCA models were trained only from complete, non-verification collection sessions. The training implementation required at least two sessions and verified that the participant identifier, experiment identifier, configuration hash, sampling rate, channels, candidate frequency order, phase offsets, epoch timing, and notch-filter configuration were compatible across sessions.

EEG epochs were obtained from frame-confirmed stimulus_onset and stimulus_offset records. The default FBTDCA configuration extracted a 3.5 s epoch beginning 0.25 s after confirmed stimulus onset. Incomplete windows were excluded and recorded with an explicit reason. A 60 Hz IIR notch filter with quality factor 30 was applied where enabled.

The reference processor used the BRAInDA implementation of filter-bank task-discriminant component analysis (FBTDCA), combining task-discriminant component analysis, established filter-bank decomposition principles, and related spatial-filtering concepts for SSVEP recognition [13,14,15,16]. The exact BRAInDA source revision was pinned in the repository to preserve software provenance. The stored algorithm contract specified five reference harmonics, three filter-bank bands, eight components, zero padding, and filter weights of $i^{-1.25}+0.25$ for filter-bank index *i*.

The reference route did not use a separate handcrafted feature vector. After epoch extraction and optional notch filtering, the FBTDCA implementation applies filter-bank decomposition and task-discriminant spatial projection to form the discriminative representation used for frequency classification. In this manuscript, that route is included to document reproducible processing provenance rather than to claim a new feature extraction or classification method.

Leave-one-session-out validation was used during training, with each collection session serving once as the held-out fold. Accuracy, balanced accuracy, confusion matrices, normalized confusion matrices, per-class recall, and fold-specific results were generated by the training workflow. The final participant-specific model was then trained on all eligible collection epochs. Model metadata preserved the participant identifier, acquisition rate, channel order, candidate frequencies, phase offsets, processing-window definition, notch settings, preprocessing description, algorithm settings, BRAInDA revision, package versions, and identifiers with configuration hashes of the source sessions.

Online verification was recorded separately at the trial level, including the target and predicted frequencies, model scores, processing status, latency, and diagnostic information. The corresponding summary distinguished scored, failed, unscored, and missing trials and reported overall accuracy, balanced accuracy, confusion matrices, per-class performance, and processing latency statistics. These outputs document the processing path and its provenance; they were not used to make population-level performance claims in this paper.

### 2.10. Independent Photoresistor Measurement System

Physical visual stimulation was evaluated using the independent ssvep-frequency-test subsystem. An Arduino equipped with a photoresistor sampled the sensor on analog input A0 at a fixed nominal cadence of 400 Hz and transmitted comma-separated records containing sample_index, device_time_us, and light_amp at 115200 baud.

The photosensor recorder ran independently of the presentation application. Each sample received host monotonic and wall-clock receipt timestamps. Gaps in the Arduino sample index were used to identify missing serial records, while the Arduino microsecond counter provided the primary time base for estimating sample spacing and stimulus frequency. Using device time in this way reduced sensitivity to buffering and bursty serial delivery on the host.

The first 100 received samples were discarded by default as serial and device warm-up. The resulting light_amp.csv contained host monotonic time, host wall-clock time, light amplitude, device sample index, and device microsecond time. A separate photosensor_sync.csv preserved recorder start, warm-up-end, and termination synchronization records.

**Figure 3:**
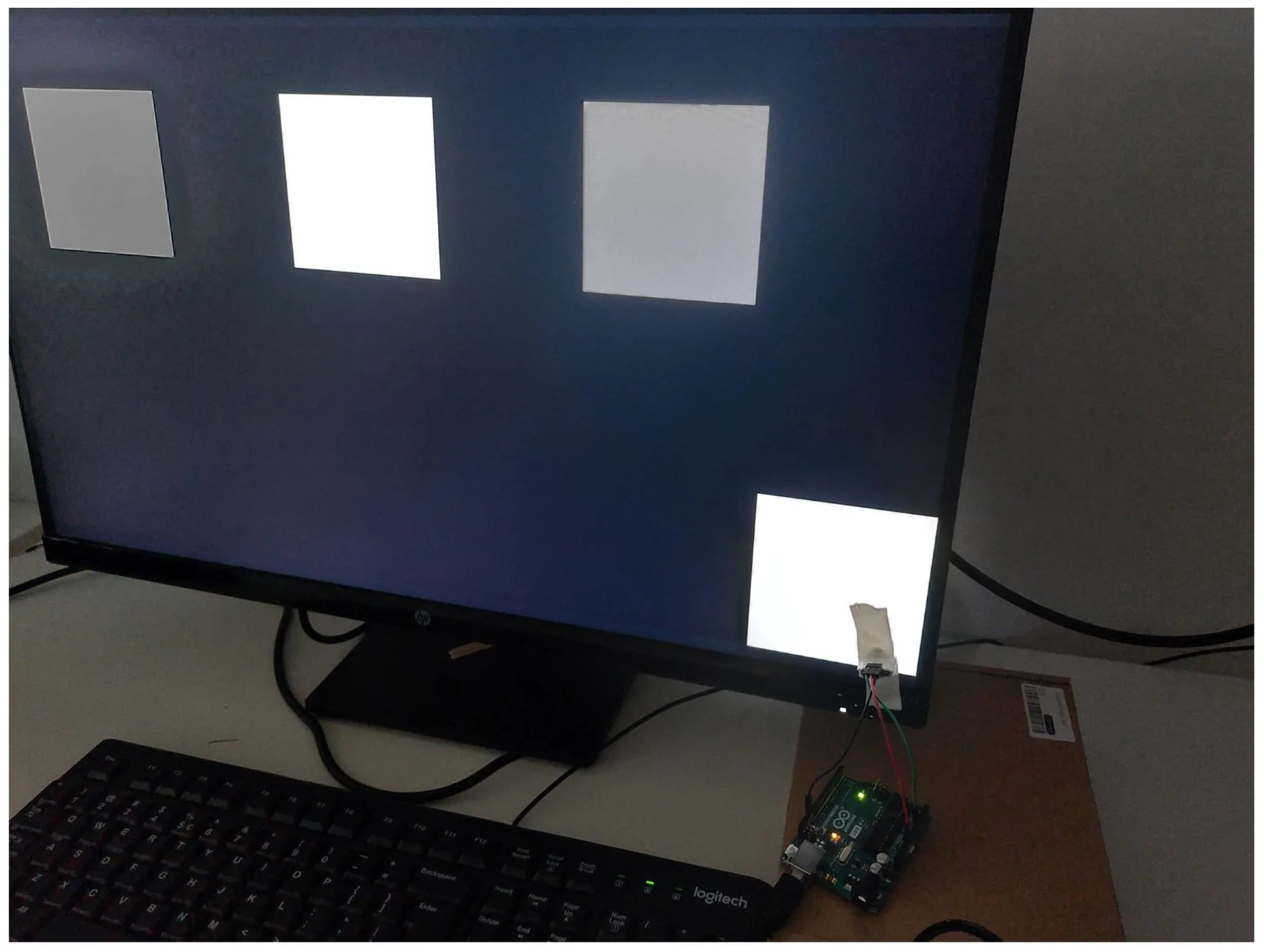
Physical setup used for independent SSVEP stimulus validation. A photoresistor was fixed over the measured bottom-right 400 × 400-pixel square on the HP P27q G4 display and connected to an Arduino Uno sampling analog input A0 at a nominal 400 Hz. The optical recorder operated independently of the SSVEP presentation process. No human participant was involved in the validation series.

The photoresistor was fixed directly over the measured 400 × 400-pixel rectangle in the bottom-right corner of the display and connected to analog input A0 of an Arduino Uno. The repository firmware sampled at a nominal 400 Hz and streamed at 115,200 baud. Room lighting remained on during the formal series, and no optical shroud or blackout material was used. Even under this simple setup, the recordings showed clear ON/OFF contrast without saturation at the 10-bit ADC ceiling. The monitor, resolution, refresh-rate setting, Windows scaling, GPU, sensor position, cabling, Arduino configuration, and frozen software revision were unchanged throughout the formal series. Figure 3 shows the physical arrangement.

### 2.11. Optical Signal Alignment and Frequency Analysis

For analysis, frame-confirmed stimulus events were read from the SSVEP session together with the corresponding configured stimulus metadata. Stimulus onset and offset records were paired by presentation_id. Only onset events explicitly marked confirmed_by_frame_swap = true were accepted for physical analysis windows.

When device timing was available, the Arduino microsecond counter was unwrapped to obtain a monotonic device-time sequence. The sampling rate was calculated from the median device-time difference between consecutive samples. Missing samples were estimated from discontinuities in the Arduino sample index.

Because USB and operating-system buffering can cause samples to reach the host in bursts, the optical signal was retimed onto a uniform device-time grid prior to spectral analysis. Host monotonic times were used to map the independent optical stream onto the monotonic clock domain of the experiment. For legacy recordings lacking device timing, the software instead estimated effective sampling rate from the active host-time span after excluding long serial stalls.

Trial windows were extracted from the retimed optical recording using the frame-confirmed experimental timestamps plus a constant 0.07 s alignment latency, which was the analyzer default for all formal validation runs. This latency shifts the temporal placement of the analysis window but does not affect frequency calibration from the Arduino device clock.

Each trial segment was demeaned and multiplied by a Hann window before frequency estimation. A one-sided spectrum was computed with the real-valued fast Fourier transform, and spectral power was taken as the squared magnitude of the transform. The detected frequency was the maximum-power bin between 1 and 40 Hz. No sub-bin peak interpolation was used. With approximately 10 s of optical data sampled near 400 Hz, the effective Fourier-bin spacing was about 0.1 Hz. Individual detected frequencies were, therefore, quantized to the FFT grid. Aggregate means and absolute-error statistics are retained at four decimal places to reproduce the exact arithmetic summaries of the trial-level values; this numerical reporting precision must not be interpreted as physical frequency resolution at the 10^−4^ Hz scale. Small differences between condition means were treated descriptively rather than as millihertz-scale physical changes.

For each trial, the signed frequency error was calculated as

(1) $$\Delta \;f=f_{detected}-f_{target},$$

and the absolute frequency error as

(2) $$\left|\Delta \;f\right|=\left|f_{detected}-f_{target}\right|.$$

A trial passed the predefined frequency criterion when

(3) $$\left|\Delta \;f\right|\leq 0.25\mathrm{Hz}.$$

Equation (3) applies the acceptance criterion to the absolute error |Δf| defined in Equation (2). The ±0.25 Hz tolerance was defined before the formal validation series and was not changed after the results were observed. It served as a technical quality-control threshold for detecting a material mismatch between configured and emitted dominant frequency; it was not intended as a criterion for discriminating adjacent SSVEP classes. In particular, passing this threshold does not imply physical separability of targets spaced more closely than 0.25 Hz, as may occur in dense multi-target SSVEP designs.

Optical contrast was calculated from the 5th and 95th percentiles of light amplitude. The software required a dynamic range of at least 40 ADC units, approximately 4% of the nominal 10-bit Arduino range. This check was intended to flag recordings affected by saturation, poor sensor placement, or insufficient exposure to the flashing region.

The analysis also examined the third and fifth harmonic bins expected from a square-wave stimulus. Harmonic presence was assessed relative to the fundamental amplitude using the predefined implementation threshold. These values were used as diagnostics rather than primary measures of frequency accuracy.

### 2.12. Optical Pulse-Width and Timing Diagnostics

Frequency accuracy and local timing regularity were assessed separately. To identify unusually long ON or OFF intervals, the optical waveform was binarized with a threshold midway between its 20th and 80th percentile amplitudes. Consecutive identical binary samples were then grouped into runs.

For a target frequency f and photoresistor sampling rate $f_{s}$, the nominal half-cycle duration in samples was calculated as

(4) $$w_{nominal}=\frac{f_{s}}{2f}.$$

In Equation (4), *f_s_* denotes the effective photoresistor sampling rate in samples per second, f is the target stimulus frequency in hertz, and *w_nominal_* is the expected ON or OFF half-cycle width in samples. The factor of two follows from the 0.5 duty cycle used in the formal validation profiles, for which one full stimulus cycle contains one ON and one OFF half-cycle.

The implementation classified an ON or OFF run as an optical pulse-width anomaly when its duration exceeded 1.5 times the nominal half-cycle width. Although the internal output field is named ‘frame_skips’, the manuscript uses the more conservative term optical pulse-width anomaly because the detector operates on thresholded optical half-cycles rather than graphics-driver frame counters. The anomaly count and the number of detected transitions were retained for each trial.

Wall-clock and monotonic alignment were also compared for individual trial onsets. For each onset, the nearest optical sample in wall-clock time was mapped back into the host monotonic domain, and the residual relative to the experimental onset timestamp was calculated. Variation in these residuals provided a check on the stability of the mapping between the two clocks.

Frequency accuracy and pulse-width diagnostics were analyzed independently. A trial could retain the correct dominant frequency while still showing local timing irregularities; such trials were not discarded simply because they passed the ±0.25 Hz frequency criterion.

### 2.13. Physical Validation Experiments

#### 2.13.1. Experiment A: Frequency Sweep from 8 to 15 Hz

The main physical validation profile tested integer target frequencies from 8 to 15 Hz. The ascending sequence of 8, 9, 10, 11, 12, 13, 14, and 15 Hz was repeated twice in each session. Each target appeared for 10 s as a 400 × 400-pixel square in the bottom-right corner of the display.

During every measured trial, three additional 400 × 400-pixel distractor rectangles flickered simultaneously at 8.25, 11.25, and 14.25 Hz. The photoresistor remained fixed over the bottom-right target square and did not overlap the distractors.

Because Experiment A evaluated monitor output rather than EEG acquisition, it used the synthetic acquisition backend. This choice isolated the presentation and optical measurement chain from participant and EEG-hardware variability. Accordingly, the experiment tests physical SSVEP stimulus delivery and its linkage to frame-confirmed session events; it does not test end-to-end synchronization to OpenBCI samples or markers. Each session comprised 3 s of initial rest, a 1 s prestimulus interval, 10 s of stimulation, a 2 s intertrial interval, and 1 s of final rest. Stimulus order was fixed.

Three complete runs were specified before data collection. Each run contained two measurements at each of eight target frequencies, giving a planned total of 48 optical trials, provided the runs met the predefined technical validity criteria.

A run was not repeated simply because the measured frequency exceeded the ±0.25 Hz criterion or a timing anomaly was detected. Repetition was allowed only after a documented technical invalidation, such as sensor displacement, recorder failure, use of an incorrect monitor configuration, loss of the measurement file, or another procedural fault that prevented interpretation. Valid PASS and FAIL results were both retained.

For each target frequency, predefined outcomes included the number of valid measurements, detected frequency, signed and absolute frequency error, mean and median absolute error, maximum absolute error, proportion within tolerance, optical dynamic range, and number of optical pulse-width anomalies.

#### 2.13.2. Experiment B: Effect of Concurrent Stimulus Rendering

A second optical experiment tested whether simultaneous rendering of additional flickering stimuli altered the measured output of a fixed 12.75 Hz target.

The baseline profile presented a single 400 × 400-pixel 12.75 Hz square in the bottom-right corner of the display. The rendering-load profile presented the same measured 12.75 Hz square at the same location while simultaneously rendering three additional 400 × 400-pixel rectangles at 8.25, 9.75, and 14.25 Hz across the upper part of the screen. Each condition contained two 10 s target trials.

Three paired repetitions of the baseline and distractor profiles were planned in advance, yielding six baseline measurements and six measurements under rendering load.

The primary comparison was the paired change in the absolute 12.75 Hz frequency error between the baseline and distractor conditions. Optical pulse-width anomalies and signal contrast were also compared descriptively. With only three paired repetitions, the experiment was treated as a technical robustness check rather than an inferential study, and every individual observation was retained.

### 2.14. Automated Software Verification and Repository Freeze

Before the formal validation results were generated, the repository was frozen at a defined Git commit and tagged release. No code changes intended to improve the physical validation outcome were allowed after the freeze. Any later correction had to be documented and, where relevant, followed by repetition of the affected validation procedure.

The repository included automated test suites for the SSVEP application, the photosensor validation subsystem, and the imagined-speech application. The tests covered configuration validation, experiment planning, runtime behavior, acquisition interfaces, recording, signal processing, model compatibility, session validation, user interface behavior, and physical validation analysis.

The automated tests were treated as evidence that the frozen implementation behaved according to its specified software and artifact generation contracts. Passing the test suite was not treated as evidence that the framework is scientifically superior to existing BCI platforms, that it improves EEG signal quality, or that it guarantees reproducibility across laboratories and hardware configurations.

All automated tests relevant to this paper were rerun from frozen commit 34732475ab927c2fd4913fb22bf7c2f2f83e274b, tagged ‘bci-reproducibility-paper-v1.0.0-rc2’, under Python 3.11.1. The ‘ssvep-bci’ suite contained 45 tests, and ‘ssvep-frequency-test’ contained 16. Complete JUnit XML and console outputs were retained. The imagined-speech application was excluded from the formal evidence package because it was not evaluated in this study.

The dependency environment was archived with the evidence package. Key versions were NumPy 2.4.6, SciPy 1.17.1, scikit-learn 1.9.1, PySide6 6.11.2, Pydantic 2.13.5, Joblib 1.6.0, BrainFlow 5.22.2, pylsl 1.18.2, pandas 3.0.5, Matplotlib 3.11.1, and pytest 8.4.2. The frozen repository contained ‘ssvep-bci’ 0.1.0 and ‘ssvep-photosensor-check’ 0.3.0.

### 2.15. Statistical and Descriptive Analysis

Because this study was a technical validation, the analysis emphasized reproducibility and measurement error rather than population inference.

Every valid optical trial from the 8–15 Hz sweep was retained as an individual observation. The results were summarized by target frequency using detected frequency, signed error, absolute error, mean, median, range, and maximum absolute error. The proportion within the predefined ±0.25 Hz criterion was calculated for each target and for the full set of trials. Pulse-width anomaly counts and optical signal quality measures were reported alongside frequency accuracy and were not used to exclude otherwise valid measurements.

For the 12.75 Hz baseline-versus-distractor experiment, measurements were paired by repetition and within-run trial number. Detected frequency, absolute frequency error, and pulse-width anomaly counts were reported for each trial and summarized by condition. Given the three planned paired repetitions, interpretation focused on the magnitude and consistency of paired differences rather than asymptotic hypothesis testing.

Automated software verification was summarized as the numbers of executed, passed, failed, and skipped tests. Session integrity evidence was reported descriptively from the available artifact and provenance checks.

No human-participant EEG data or classification results were analyzed as empirical evidence in this study.

During manuscript preparation, J.P. used ChatGPT (GPT-5.6 Sol; OpenAI, San Francisco, CA, USA) to assist with manuscript structuring, language revision, and figure development. It was not used to generate experimental data. All numerical results, technical descriptions, and interpretations were reviewed and verified by J.P. against the underlying repository and validation records.

## 3. Results

The frozen validation package contained the repository-freeze record, software environment capture, automated-test logs, three complete 8–15 Hz sweeps, three paired 12.75 Hz baseline and distractor repetitions, raw optical recordings, per-run summaries, aggregate tables, and a run and exclusion log. All formal runs were technically valid, and no valid failure or timing anomaly was removed from the analysis. The complete validation evidence package is provided as Supplementary File S1.

### 3.1. Frozen Software Release and Automated Verification

The repository was frozen at commit 34732475ab927c2fd4913fb22bf7c2f2f83e274b and annotated tag ‘bci-reproducibility-paper-v1.0.0-rc2’. The working tree was clean at freeze time, and the tag was confirmed on the remote repository. All 61 tests relevant to this paper passed (Table 3): 45/45 for ‘ssvep-bci’ and 16/16 for ‘ssvep-frequency-test’. One non-failing Matplotlib ‘FigureCanvasAgg’ warning occurred during a synthetic FBTDCA notebook smoke test; the frequency validation suite produced no warnings. These results verify that the frozen implementation behaved as specified under the tested software conditions; they are not treated as evidence that the workflow is scientifically superior to existing BCI systems, improves EEG data quality, or guarantees reproducibility outside the tested configuration.

### 3.2. Session Integrity and Reproducibility Evidence

Automated verification covered complete synthetic-session recording, required raw-session structure, frame-acknowledged stimulus intervals, and manifested and checksummed verification artifacts, and all of these tests passed. Each formal optical run also retained a provenance record with the run identity, experimental profile, source session identifier and path, UTC creation time, frozen revision, and terminal status. All nine formal validation sessions were recorded as complete. The archive contained raw ‘light_amp.csv’ and ‘photosensor_sync.csv’ files, per-run ‘summary.csv’ files, and aggregate ‘all_sweeps.csv’ and ‘all_load_tests.csv’ tables. Because the complete underlying SSVEP session directories were referenced but not included in the archived handoff, the integrity result reported here relies on the frozen automated tests and retained run-level provenance rather than a separate post hoc ‘validate-session’ rerun for each formal session.

### 3.3. Physical Frequency Accuracy Across 8–15 Hz

**Figure 4:**
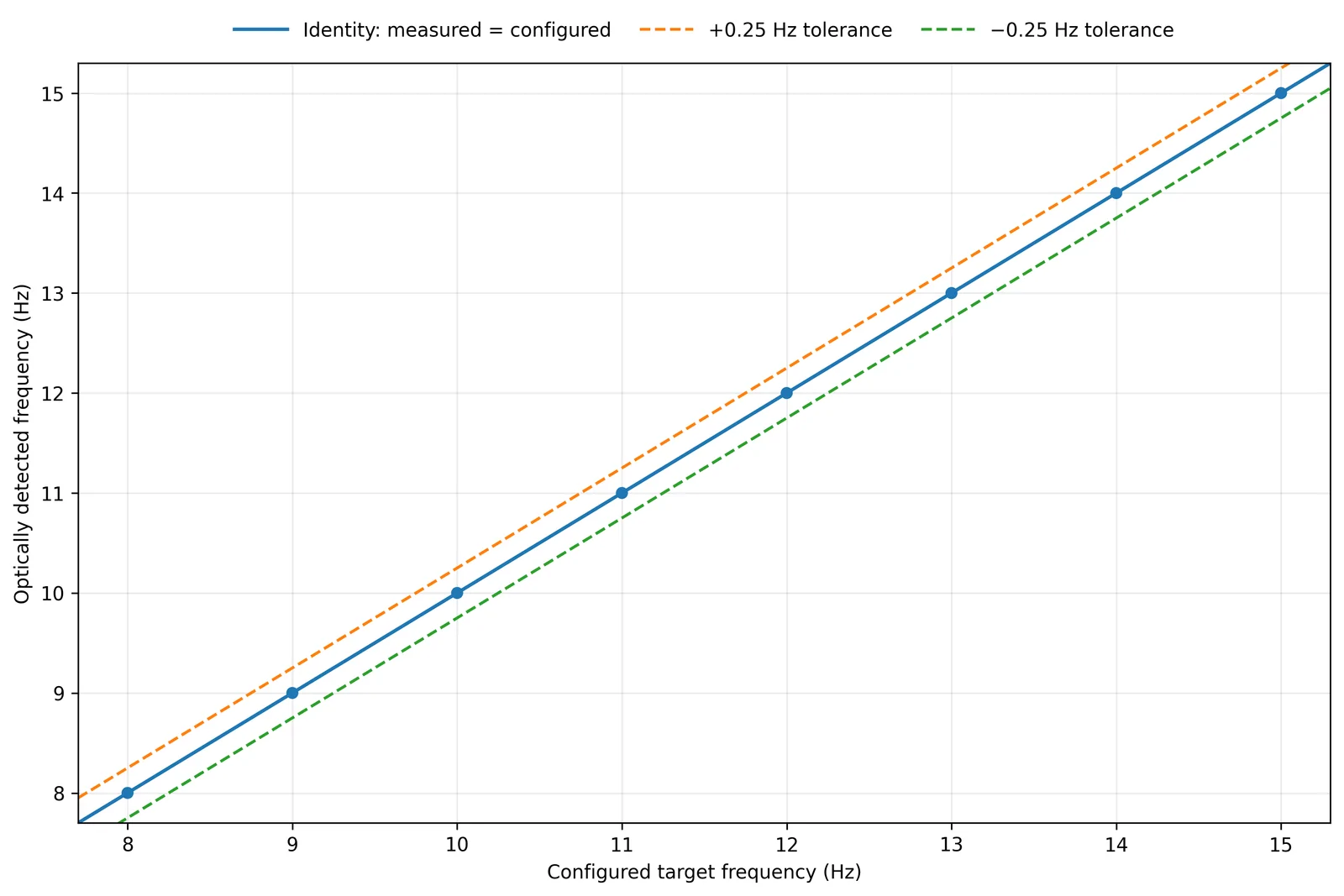
Configured target frequency versus optically detected frequency for all 48 valid measurements from the three 8–15 Hz sweep runs. The solid identity line represents perfect agreement; the orange and green dashed lines denote the +0.25 Hz and −0.25 Hz tolerance bounds, respectively.. All measurements remained within tolerance.

All three planned 8–15 Hz sweep runs were technically valid and yielded 48 analyzable optical trials, six at each target frequency. Every measurement fell within the predefined ±0.25 Hz criterion. Across the 48 trials, the mean absolute frequency error was 0.0345 Hz, the median absolute error was 0.0313 Hz, and the maximum absolute error was 0.0668 Hz. Table 4 gives the frequency-specific values, and Figure 4 plots every configured-versus-detected measurement against the identity and tolerance lines. Optical contrast remained high, with per-trial 5th-to-95th-percentile amplitude differences of 536.4–656.0 ADC units. The four-decimal error values are arithmetic summaries of discrete FFT-bin estimates. Because the approximately 10 s windows provide about 0.1 Hz spectral-bin spacing and no sub-bin interpolation was used, these aggregate values must not be interpreted as indicating measurement resolution at the 10^−4^ Hz level.

### 3.4. Frequency Error and Frame Stability

Accurate dominant frequency did not always coincide with locally regular timing. Although every trial passed the frequency criterion, the pulse-width detector identified 175 anomalies across the sweep series. Counts were strongly frequency-dependent: 0 at 8 Hz; three each at 9, 10, 11, and 12 Hz; 33 at 13 Hz; 124 at 14 Hz; and six at 15 Hz. The 13 and 14 Hz targets accounted for 157 of 175 anomalies (89.7%) while still retaining dominant-frequency estimates within the predefined tolerance. Because stimulus phase was generated from elapsed host monotonic time, this dissociation is partly expected by design: a long-window Fourier estimate can remain centered near the configured frequency even when individual optical half-cycles are locally irregular. The result is, therefore, not interpreted as a new display-timing mechanism. Rather, it shows that dominant-frequency agreement alone does not rule out local physical timing irregularity in the tested setup.

### 3.5. Effect of Simultaneous Rendering

**Figure 5:**
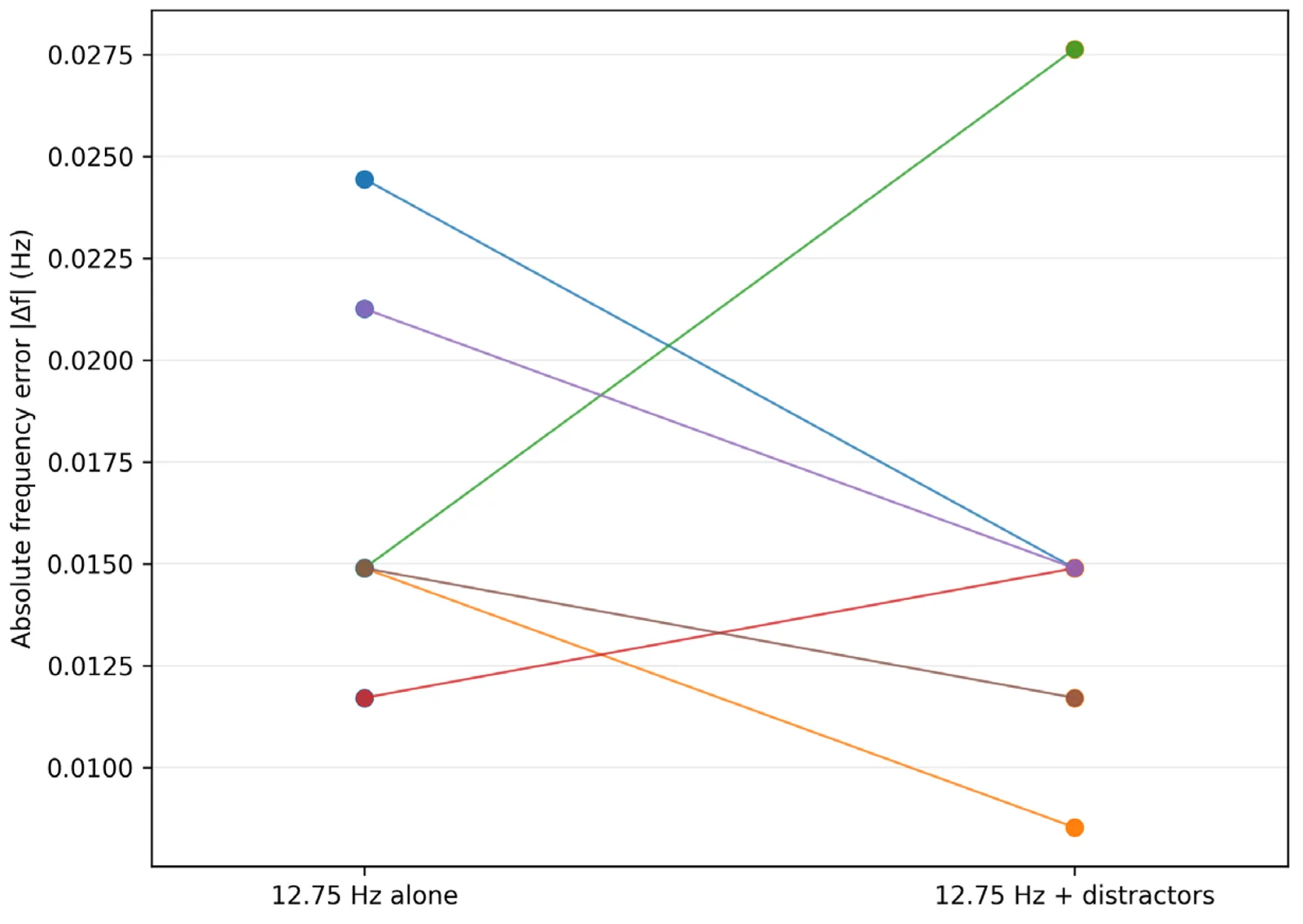
Paired absolute frequency error for the 12.75 Hz target presented alone and with three simultaneous distractors. Each line connects corresponding trial numbers within the same repetition. All 12 measurements satisfied the predefined ±0.25 Hz criterion. Aggregate optical pulse-width anomaly counts were 1 for the baseline condition and 27 with distractors.

All 12 measurements in the 12.75 Hz rendering-load experiment met the ±0.25 Hz criterion. The six baseline trials had a mean absolute error of 0.0170 Hz (median: 0.0149 Hz; maximum: 0.0245 Hz), compared with 0.0154 Hz (median: 0.0149 Hz; maximum: 0.0276 Hz) for the six trials with three simultaneous distractors. Figure 5 shows the paired trial-level results. Because the FFT-bin spacing was approximately 0.1 Hz, the difference between the two arithmetic means is below the measurement resolution and is not interpreted as a physical improvement or deterioration under load. Pulse-width anomalies increased from one in the baseline condition to 27 with distractors. This increase was uneven across repetitions: load-01 showed no anomalies under distractor load, while load-02 showed 10, and load-03 showed 17. Concurrent rendering, therefore, left the recoverable dominant 12.75 Hz frequency essentially unchanged within the predefined criterion, while local optical timing irregularities were more frequent in two of the three repetitions. This comparison remains descriptive because only three paired repetitions were performed.

The raw optical recordings also showed stable sampling. Arduino sample indices were contiguous in all nine files, with no missing samples. The median device-time increment was 2500 microseconds in every recording, corresponding to 400 Hz, and individual increments ranged from 2488 to 2512 microseconds. Each 10 s sweep trial contained approximately 4007–4013 optical samples. In the sweep series, the fifth-percentile amplitude was approximately 255–265 ADC units and the 95th percentile approximately 798–912 ADC units, with no ceiling saturation. These diagnostics were consistent with adequate signal contrast and sampling continuity for the frequency validation analysis.

## 4. Discussion

### 4.1. Reproducibility as an Experimental-Chain Property

This study contributes an experimentally evaluated audit structure rather than a new SSVEP decoder or a new individual software primitive. Machine-readable configuration, synchronized data streams, software integrity checks, and independent optical stimulus measurement are established practices. The contribution examined here lies in keeping their evidence roles distinct while linking them within one session record: configured experimental intent, runtime frame acknowledgement, acquisition provenance, and independently measured physical display output.

This emphasis follows directly from a broader reproducibility problem in EEG-BCI research. Dataset fragmentation, heterogeneous acquisition conditions, incomplete metadata and preprocessing descriptions, and subject- and session-related variability continue to limit comparison and reuse across studies [2]. Making classifier code available is only one part of reproducibility. If the conditions under which a neural signal was generated are poorly represented, later analyses remain constrained by uncertainty about the original experiment.

The workflow captures that context at the time of data generation. Configuration is retained with the session, trial and presentation identities are carried into runtime events, raw signals remain separate from derived results, and acquisition state is logged explicitly. These measures cannot by themselves guarantee cross-laboratory reproducibility. Their narrower purpose is to reduce the amount of experimental context that must later be reconstructed from undocumented assumptions and to make disagreements between evidence layers inspectable.

This role is complementary to established BCI infrastructures. BCI2000 and OpenViBE provide mature experimental and online architectures [5,6], LSL supports synchronized multimodal streaming [7], and more recent frameworks integrate acquisition, stimulus presentation, markers, and experiment control on open platforms [17,18,25]. EEG-BIDS standardizes the organization and metadata of reusable EEG datasets [3], while MOABB and its recent extensions support reproducible algorithm benchmarking across multiple datasets and pipelines [4,19,20]. Reproducibility studies across EEG software environments further show that apparently equivalent processing choices can produce divergent outcomes when implementation details are insufficiently controlled [21]. The present work does not replace these systems; it addresses the narrower problem of preserving and cross-checking the relation between intended protocol, runtime execution, acquisition provenance, and selected physical stimulus properties within a single experimental record.

Accordingly, the scientific claim is not that any one component of the workflow is new. The evaluated contribution is the explicit cross-layer audit trail and the empirical comparison between software-side execution evidence and independently measured display output under a frozen implementation. This comparison revealed that a stimulus can satisfy the predefined dominant-frequency criterion while still exhibiting local optical pulse-width irregularities. The inference is intentionally limited: the observation supports the need to preserve more than a single frequency estimate when auditing the tested SSVEP presentation chain, but it does not demonstrate universal superiority over existing BCI frameworks. Accordingly, the contribution is not simply the co-location of existing mechanisms, but their use as independent evidence layers whose agreement and disagreement can be evaluated experimentally.

### 4.2. Frame-Confirmed Events and Physical Stimulus Validation Address Different Questions

Frame-confirmed events and physical validation answer different questions and should not be treated as interchangeable evidence.

Frame confirmation establishes that the requested visual state reached the OpenGL frame-swap boundary. Compared with a trigger generated when presentation is merely requested, it removes one source of software-side ambiguity by moving the event timestamp to a later, renderer-confirmed boundary. It does not eliminate display latency, quantify the time from emitted luminance to an EEG sample, or guarantee synchronization through the physical OpenBCI transport path; those quantities were not measured in the formal validation.

The frame-swap event cannot establish the exact luminance waveform emitted by the monitor. Display electronics, refresh behavior, repeated or dropped frames, and other hardware-dependent effects occur downstream of the software acknowledgement. Earlier timing studies reached a similar conclusion: laboratory presentation systems can be highly precise, but the actual hardware and stimulus configuration still require validation [8,9]. More recent frequency-tagging and SSVEP work likewise treats display refresh behavior and independent optical verification as material experimental considerations [22,23,24].

The photoresistor provides that independent physical check by measuring emitted luminance directly. Keeping the optical record separate from the frame-swap event preserves two different kinds of evidence: one describes runtime semantics inside the software stack, and the other tests whether a physically meaningful stimulus property was realized at the display.

For SSVEP experiments, this distinction is especially relevant because stimulation frequency is part of the experimental condition itself. CCA, filter-bank methods, and task-discriminant component analysis all rely on the relationship between EEG activity and the stimulus frequency or its harmonics [10,13,14,15]. A mismatch between configured and emitted frequency is experimental information, not merely an implementation detail.

All 48 sweep measurements met the predefined frequency criterion, and the maximum absolute error was 0.0668 Hz. Within the tested configuration, the long-window FFT, therefore, recovered the configured dominant frequencies within the predefined quality-control tolerance. This result should be interpreted in light of the stimulus generation method: because phase is recomputed from elapsed monotonic time, occasional local frame or pulse irregularities need not displace the dominant spectral peak over a 10 s window. The concentration of optical pulse-width anomalies at 13–14 Hz, therefore, does not contradict the frequency result. Instead, it shows why frequency-only validation is incomplete for this implementation. The ±0.25 Hz threshold is likewise a technical acceptance criterion for detecting gross stimulus-frequency mismatch, not a claim that targets separated by 0.25 Hz or less would be experimentally or physiologically distinguishable. Dense SSVEP designs require validation criteria appropriate to their own target spacing and display configuration.

### 4.3. Provenance Before Algorithm Benchmarking

BCI performance analyses commonly reduce an experiment to EEG epochs, class labels, and outcome metrics. That abstraction is necessary for algorithm evaluation, but it can obscure how the labels and epochs were generated. The present study focuses on those preceding layers rather than claiming a new benchmark. This is complementary to MOABB-style comparisons, which standardize downstream evaluation across heterogeneous datasets and pipelines [4,19,20], and to EEG-BIDS, which standardizes the organization and description of reusable EEG datasets [3].

The configuration records the intended protocol, the compiled plan records the execution sequence, runtime events retain the timing and identity of protocol transitions, and raw EEG preserves acquisition-level timestamps and channel values. Acquisition metadata add the backend, channel configuration, sampling rate, and sample count, while marker and health records capture runtime information that would otherwise be lost. Optional classifier and verification outputs remain separate from the primary recording.

This organization reduces reliance on manually reconstructed semantics and makes cross-file inconsistencies detectable. For example, the validator can compare the EEG sample count with acquisition metadata, verify contiguous sample indices, parse every runtime event, and check SHA-256 digests when they are available. These functions are engineering mechanisms, but their scientific role in this study is to preserve the provenance needed to interpret later measurements and analyses rather than to serve as evidence of algorithmic superiority.

These mechanisms are not intended to replace data standards such as EEG-BIDS [3] or integrated experiment platforms such as BCI2000, OpenViBE, the OpenBCI framework, BCI-HIL, or NeuronLab [5,6,17,18,25]. Those systems address overlapping but broader needs in acquisition, experiment control, streaming, and data organization. The present workflow instead makes the configured protocol, runtime acknowledgements, acquisition artifacts, and physical stimulus measurement explicitly cross-referable within the same session-level audit structure.

The same distinction applies to MOABB [4,19,20]. MOABB improves reproducibility at the benchmarking stage by applying consistent pipelines to heterogeneous BCI datasets, whereas this study examines how provenance and physically verifiable conditions can be preserved before such downstream benchmarking occurs. The two approaches, therefore, address different points in the reproducibility chain rather than competing alternatives.

### 4.4. Implications for SSVEP Experimentation

SSVEP offers a useful validation case because stimulus frequency, one of the key experimental variables, can be measured independently of the EEG.

This allows three layers to be compared directly: the frequency specified in the protocol, the runtime event produced when the visual state reaches the frame-swap boundary, and the frequency recovered from emitted luminance. Agreement among these layers supports the internal consistency of the tested stimulus record; disagreement would expose an inconsistency requiring investigation. Because the formal optical sessions used the synthetic acquisition backend, however, this comparison does not extend to end-to-end EEG-marker latency or packet loss on the physical OpenBCI link.

The rendering-load experiment adds a narrower technical check. By measuring the same 12.75 Hz target both alone and with simultaneous distractors, it tests whether the final visual workload materially changes the physical output of that target. It is not intended to establish a universal limit for the graphics subsystem.

Under the tested configuration, the three distractors did not produce a resolvable change in the dominant 12.75 Hz frequency. All measurements remained within tolerance, and the reported mean absolute errors were 0.0170 and 0.0154 Hz for the single-stimulus and distractor conditions, respectively. These means are arithmetic summaries of FFT-bin estimates and should not be read as millihertz-scale measurement precision; with approximately 0.1 Hz bin spacing, the difference is below the resolution of the analysis. Pulse-width anomalies were more sensitive to the rendering condition, rising from one across the six baseline trials to 27 across the six distractor trials. The increase was uneven across repetitions, so the appropriate conclusion is limited to the tested setup: concurrent rendering did not cause a resolvable dominant-frequency shift, but local timing irregularities occurred more often in two runs.

The small number of repeated bench measurements limits this comparison to descriptive interpretation. Its purpose is to determine whether an obvious load-related effect is visible under one frozen configuration, not to establish a general performance bound for graphics hardware, consumer monitors, or low-cost BCI systems.

The FBTDCA route was not evaluated as a competing classifier. It is included to show how provenance-rich acquisition can continue into participant-specific analysis. Pinning the BRAInDA revision and storing model metadata keeps the resulting outputs tied to the source sessions, configuration, channel order, frequency definitions, preprocessing settings, and software version [13,14,15,16].

### 4.5. Low-Cost Hardware and Reproducible Experimental Practice

OpenBCI and Arduino were used because they make EEG-BCI experimentation and independent physical validation accessible without specialized proprietary hardware. OpenBCI Cyton has been used in contemporary visual EEG and SSVEP studies [11], and its eight-channel, 250 Hz configuration is suitable for many prototyping and research tasks.

The results should not be read as evidence that low-cost equipment is equivalent to high-density or research-grade EEG systems; that comparison was outside the study design. The relevant point is methodological. When an experiment is assembled from a consumer display, a general-purpose computer, open-source software, and accessible biosignal hardware, its behavior should be documented and checked rather than assumed from nominal specifications.

The same argument applies to the optical subsystem. A photoresistor and Arduino do not replace calibrated photometric equipment, but they provide an independent path for confirming the expected periodic luminance modulation, estimating its dominant frequency, and flagging obvious pulse-width irregularities. That level of measurement is appropriate to the validation question addressed here.

The optical acquisition was stable enough for this purpose. No Arduino sample-index gaps occurred in the nine raw recordings. The median device interval was 2500 microseconds (400 Hz), and optical contrast remained far above the 40-ADC-unit minimum. The 5th-to-95th-percentile amplitude difference was 536.4–656.0 ADC units in the sweep series and approximately 559.5–564.0 ADC units in the 12.75 Hz load series. These values support adequate sensor placement and contrast, while stopping short of any claim of calibrated photometric accuracy.

### 4.6. Relationship with Dataset Interoperability and Reuse

This study does not introduce an interoperability standard, ontology, or cross-dataset semantic representation, and the present data do not demonstrate interoperability across independent datasets. Its relevance to reuse is narrower: EEG data become harder to interpret when the conditions that produced them are incompletely described or when configured conditions cannot be linked to runtime and physical evidence.

Peksa’s recent systematic review identified dataset fragmentation, reproducibility limitations, heterogeneous experiment descriptions, and cognitive and user variability as connected barriers to broader reuse of EEG-BCI data [2]. The contribution here addresses one part of that problem by retaining detailed provenance within an individual experimental workflow.

Internal consistency within one experiment is not the same as semantic interoperability across datasets. Two well-documented datasets may still differ in task semantics, event definitions, sensor layouts, preprocessing histories, or descriptions of participant state. At the same time, semantic alignment becomes substantially harder when the original conditions were never recorded explicitly.

The framework is, therefore, best viewed as supporting one enabling condition for later interoperability work rather than as an interoperability solution. Even a simple SSVEP label such as 13 Hz encompasses several distinct elements: the configured target frequency, the visual object’s identity and location, presentation timing, the frequency emitted by the display, the EEG montage and acquisition settings, and the processing context used to interpret the response. Preserving these distinctions at acquisition time can make later normalization or semantic mapping better informed, but the present study does not test whether such mapping succeeds across datasets.

A subsequent step would be to map this session-level provenance into formal semantic representations and standardized data structures and then test those mappings across independently collected BCI datasets. That problem is outside the scope of the present paper and requires separate evidence.

### 4.7. Limitations

The empirical validation has several limitations. First, it concerns one software implementation and one primary host, display, photoresistor, Arduino, and EEG-device configuration. The measured stimulus behavior should not be generalized automatically to other monitors, graphics adapters, operating systems, refresh-rate settings, computers, photosensors, or EEG acquisition hardware. No claim of reproducibility across different low-cost hardware configurations is made; this study characterizes the tested configuration and a procedure for auditing it.

Second, the physical validation is specific to visual SSVEP stimulation. Other paradigms may depend on different physical or behavioral variables and would need different validation procedures. The optical method used here does not validate auditory, tactile, motor-imagery, cognitive-state, or imagined-speech experiments.

Third, no large prospective human cohort was used as the main empirical basis. The results do not establish population-level SSVEP decoding accuracy, cross-participant generalization, or classifier superiority. FBTDCA and online verification are included as reproducible processing paths, not as new decoding contributions.

Fourth, frame-swap confirmation is not a direct measurement of optical onset. It marks a boundary within the rendering process and should be interpreted together with external physical measurement. In addition, all formal optical sessions used the synthetic acquisition backend. Consequently, this study does not empirically quantify end-to-end event-to-EEG alignment, OpenBCI marker latency, USB or wireless transport latency, or packet loss on the physical EEG link.

Fifth, the photoresistor system has its own measurement limits. Accuracy depends on Arduino sampling, photoresistor response, sensor placement, contrast, timing alignment, and the frequency estimation procedure. The nominal 400 Hz sampling rate was sufficient for the 8–15 Hz stimuli studied here, but approximately 10 s FFT windows provide only about 0.1 Hz bin spacing, and no sub-bin interpolation was used. The four-decimal aggregate error values, therefore, summarize discrete-bin estimates and do not represent a four-decimal physical frequency resolution. Higher-frequency, more densely spaced, or more complex visual stimulation would require separate validation of the measurement method and acceptance criterion.

Sixth, the pulse-width detector is a threshold-based timing diagnostic, not a direct counter of dropped graphics frames and not a complete description of display timing. The internal field name ‘frame_skips’ should not be read as graphics-driver evidence. Absolute photometric onset latency, pixel-response dynamics, and sub-frame behavior would require more specialized instrumentation.

Seventh, the framework does not currently export directly to EEG-BIDS and does not implement a semantic ontology or a cross-dataset interoperability layer. Those remain possible extensions rather than capabilities demonstrated here.

Eighth, although the repository includes an imagined-speech acquisition application, this paper provides no evidence about imagined-speech decoding or about using SSVEP calibration to predict imagined-speech signal quality. The two paradigms should not be treated as physiologically equivalent.

Finally, automated tests establish expected software behavior under defined conditions; they do not by themselves establish scientific novelty, superior reproducibility, improved EEG data quality, or the correctness of every experiment built with the software. Reproducibility still depends on sound protocol design, correct hardware setup, operator practice, participant management where relevant, transparent reporting of deviations, and external validation where the experimental variable has a physical realization.

These limitations define the scope of the claims. Under the evaluated configuration, this study shows that configured intent, runtime acknowledgement, session provenance, software integrity evidence, and independently measured SSVEP output can be kept separately inspectable and cross-referenced. It does not establish algorithmic superiority, population-level BCI performance, cross-hardware equivalence, end-to-end EEG synchronization accuracy, or semantic interoperability.

## 5. Conclusions

This study evaluated a reproducibility-first workflow for EEG-BCI experimentation in which experimental configuration, deterministic session planning, frame-confirmed runtime events, acquisition provenance, software verification, and independent physical stimulus measurement remain separately auditable. All 61 automated tests passed for the frozen implementation, providing evidence of expected software behavior under the tested conditions. In the optical validation, all 48 measurements across 8–15 Hz and all 12 measurements in the 12.75 Hz rendering-load experiment met the predefined ±0.25 Hz quality-control criterion. The reported aggregate errors are constrained by the approximately 0.1 Hz FFT-bin spacing and should not be interpreted as sub-bin measurement precision. Correct dominant-frequency recovery coexisted with local optical pulse-width irregularities, particularly at 13–14 Hz and in two rendering-load repetitions. Because stimulus phase was generated from host monotonic time, this coexistence is partly expected; its practical significance is that dominant-frequency agreement alone is insufficient to characterize local stimulus timing. The contribution is, therefore, the explicit cross-layer auditability of the tested experimental chain rather than a claim that its individual components are new. This study does not establish population-level decoding performance, cross-hardware reproducibility, end-to-end OpenBCI timing accuracy, or semantic interoperability. It provides a tested basis for preserving and comparing configuration, runtime, provenance, and physical stimulus evidence within a single EEG-BCI experimental workflow.

## Figures and Tables

**Table 1:** Reproducibility requirements and implementation mechanisms.

ID	Reproducibility Requirement	Implementation Mechanism	Auditable Evidence
R1	Explicit experimental specification	Experiments are defined in validated YAML configuration files containing experiment identity, random seed, device profile, stimuli, protocol timing, presentation parameters, output settings, processing configuration, and markers.	experiment-config.yaml; device-profile.yaml
R2	Deterministic session planning and stable experimental identity	A validated configuration is compiled into a SessionPlan; an SHA-256 configuration fingerprint and stable trial_id/presentation_id values preserve the correspondence between intended and executed trials.	session-plan.json; config_hash; plan_id; trial and presentation identifiers
R3	Runtime-confirmed stimulus events	Visual presentation is performed by a PySide6/OpenGL renderer. Stimulus onset and offset events are registered after the corresponding display frame reaches the frame-swap boundary rather than only when presentation is requested.	events.jsonl; frame-confirmed stimulus_onset and stimulus_offset events
R4	Preservation of raw acquisition independently of processing	EEG acquisition and raw-data writing are separated from optional online processing. Raw samples retain source and aligned timestamps; processing outputs are stored separately.	eeg_raw.csv; optional processing-results.jsonl
R5	Session provenance and post-write integrity checking	Session identity, configuration snapshots, acquisition metadata, marker outcomes, health information, processing outputs, and optional SHA-256 digests are retained as separate artifacts. A dedicated validator checks structural and cross-file consistency.	manifest.json; acquisition metadata and logs; checksums.sha256; validate-session result
R6	Independent verification of physically meaningful stimulus parameters	Monitor output is measured independently using a photoresistor and Arduino sampled at a nominal 400 Hz. Analysis quantifies detected frequency, frequency error, signal contrast, sampling integrity, harmonics, and optical pulse-width anomalies.	light_amp.csv; optical-analysis outputs; frequency validation summary

**Table 2:** Session artifacts and their scientific role.

Artifact	Status	Main Content	Scientific and Reproducibility Role
manifest.json	Core	Session ID, experiment ID, run mode, plan ID, configuration hash, software version, status, timestamps, and artifact list	Top-level identity and provenance record
experiment-config.yaml	Core	Effective experiment configuration used for the session	Preserves intended protocol and processing configuration
device-profile.yaml	Core	Effective acquisition device configuration	Preserves hardware/channel/acquisition context
session-plan.json	Core	Ordered compiled PlanStep sequence with trial and presentation IDs	Preserves the actual planned execution sequence
events.jsonl	Core	Structured runtime protocol events and timestamps	Preserves executed experimental semantics, including frame-confirmed stimulus events
eeg_raw.csv	Core	Sequential EEG samples, source timestamps, corrected and aligned timestamps, EEG channels, and embedded markers where available	Primary raw physiological data record
acquisition-metadata.json	Core	Backend, device profile, configured rate, channels, sample count, marker capability, and terminal status	Connects raw data to acquisition context and permits sample-count consistency checking
acquisition-markers.jsonl	Core	Marker insertion attempts, success or failure, marker codes, and timestamps	Makes event-to-hardware-marker delivery auditable
acquisition-health.jsonl	Core	Timestamped health or runtime events and severity	Preserves warnings, acquisition problems, and runtime state changes
processing-results.jsonl	Optional	Online processing results	Keeps derived processing separate from raw EEG
verification-results.jsonl	Verification mode	Trial-level target, prediction, scores, correctness, latency, and diagnostics	Preserves trial-level online verification evidence
verification-summary.json	Verification mode	Accuracy, balanced accuracy, confusion matrix, class performance, failed or missing results, latency, and model provenance	Reproducible summary of an online verification session
checksums.sha256	Optional finalized session artifact	SHA-256 digest for declared session artifacts	Detects post-write modification or corruption

**Table 3:** Automated software verification results.

Component	Executed Test	Passed	Failed	Skipped	Environment and Side Notes
ssvep-bci	45	45	0	0	Python 3.11.1; 1 warning; 11.08 s
ssvep-frequency-test	16	16	0	0	Python 3.11.1; 0 warnings; 1.30 s
Total	61	61	0	0	Frozen commit 3473247; tag rc2

**Table 4:** Physical SSVEP frequency validation results.

Target Frequency (Hz)	Valid Trials, n	Mean Detected (Hz)	Median Detected (Hz)	Mean |Δf| (Hz)	Maximum |Δf| (Hz)	Within ±0.25 Hz, n (%)	Optical Pulse-Width Anomalies
8	6	7.981	7.980	0.019	0.022	6 (100%)	0
9	6	8.978	8.978	0.022	0.025	6 (100%)	3
10	6	9.973	9.973	0.027	0.032	6 (100%)	3
11	6	10.969	10.970	0.031	0.036	6 (100%)	3
12	6	12.017	12.020	0.048	0.067	6 (100%)	3
13	6	12.996	12.968	0.043	0.061	6 (100%)	33
14	6	13.979	13.963	0.041	0.061	6 (100%)	124
15	6	14.956	14.959	0.044	0.049	6 (100%)	6
All frequencies	48	—	—	0.0345	0.0668	48 (100%)	175

All technically valid measurements were retained, and no formal sweep run was excluded. The anomaly counts in Table 4 refer to threshold-detected optical pulse-width irregularities, not direct graphics-driver frame-drop counts.

## Data Availability

The source code is available at https://github.com/JanisPeksa/BCI.git at frozen commit 34732475ab927c2fd4913fb22bf7c2f2f83e274b and tag bci-reproducibility-paper-v1.0.0-rc2. The validation evidence supporting the reported results includes automated-test logs, raw optical recordings, synchronization files, per-run summaries, aggregate CSV tables, and hardware and environment documentation.

## Supplementary Materials

The following supporting information can be downloaded at https://www.mdpi.com/article/10.3390/s26217065/s1. Supplementary File S1: Supplementary_File_S1_Validation_Evidence.zip, containing the frozen repository record, software environment capture, automated-test logs, hardware and environment documentation, raw optical recordings and synchronization files for all nine formal validation runs, per-run summaries, aggregate CSV tables, provenance records, diagnostic plots, and run and exclusion logs.

## Abbreviations

The following abbreviations are used in this manuscript:

ADC	Analog-to-digital converter
BCI	Brain–computer interface
CCA	Canonical correlation analysis
EEG	Electroencephalography
FBTDCA	Filter-bank task-discriminant component analysis
GPU	Graphics processing unit
LSL	Lab Streaming Layer
MOABB	Mother of all BCI Benchmarks
SSVEP	Steady-state visual evoked potential
TDCA	Task-discriminant component analysis
